# Kinetic Equilibrium of Dipolarization Fronts

**DOI:** 10.1038/s41598-018-35349-9

**Published:** 2018-11-21

**Authors:** Gurudas Ganguli, Chris Crabtree, Alex C. Fletcher, Erik Tejero, David Malaspina, Ian Cohen

**Affiliations:** 1 Plasma Physics Division, Naval Research Laboratory, Washington, DC 20375-5346 USA; 2 Laboratory for Atmospheric and Space Physics, University of Colorado, Boulder, CO 80303 USA; 3 Johns Hopkins University Applied Physics Laboratory, Laurel, MD 20723 USA

## Abstract

The unprecedented high-resolution data from the Magnetospheric Multi-Scale (MMS) satellites is revealing the physics of dipolarization fronts created in the aftermath of magnetic reconnection in extraordinary detail. The data shows that the fronts contain structures on small spatial scales beyond the scope of fluid framework. A new kinetic analysis, applied to MMS data here, predicts that global plasma compression produces a unique particle distribution in a narrow boundary layer with separation of electron and ion scale physics. Layer widths on the order of an ion gyro-diameter lead to an ambipolar potential across the magnetic field resulting in strongly sheared flows. Gradients along the magnetic field lines create a potential difference, which can accelerate ions and electrons into beams. These small-scale kinetic effects determine the plasma dynamics in dipolarization fronts, including the origin of the distinctive broadband emissions.

## Introduction

Dipolarization fronts (DF), sometimes called bursty bulk flows^1^, are created in the aftermath of magnetic reconnection when a stretched magnetic field snaps back towards a dipolar configuration. The high-resolution data from the Magnetospheric Multi-Scale (MMS) mission^2^ is revealing the plasma dynamics in DFs in extraordinary detail in the ion and electron gyro scales, which is beyond the scope of traditional fluid and magnetohydrodynamic (MHD) formalisms. DFs are characterized by a pressure gradient over a narrow plasma layer that is dragged with magnetic field lines. They are active regions emitting waves with broadband spectral signatures that are clearly visible to satellites and are diagnostics for the local physics in the DFs. The DFs introduce and distribute mass, momentum, and energy, transported from the heliosphere by the solar wind into the plasmasheet. They are associated with particle acceleration and energy redistribution throughout the near-Earth space environment, which makes them an important contributing factor to the near-Earth space weather. Hence, knowledge of their detailed properties is necessary for developing a predictive capability in this region. However, the physics of the DF dynamics remains obscure because fluid/MHD frameworks generally applied to address them^3,4^ ignore the important small-scale kinetic features of the narrow layers and they are not adequately resolved in the global kinetic simulations^5–8^ of the magnetotail. These narrow layers maintain strong spatial gradients in critical plasma parameters in both electron and ion gyro scales that determine the kinetic physics. Hence, the objective of this report is to discuss a Vlasov equilibrium solution that captures the vital small-scale spatial variabilities and their kinetic effects in order to quantify their measurable signatures. This equilibrium, which we match to observations, is a rigorous Vlasov solution that can be used as an initial condition to study the stability and nonlinear evolution of DFs.

The core physics of DFs is similar to that of the lobe-plasmasheet interface discussed by Romero *et al*.^9^. Important to this physics is the existence of a strongly localized ambipolar electric field transverse to the magnetic field^10–13^, which is accompanied by broadband waves^14^ and energy dissipation. While some of the properties of such boundary layers transverse to the magnetic field have been studied in the context of the plasmasheet-lobe interface^9,15^, the consequences of the equilibrium parallel to the magnetic field have not. For example, the origin of the parallel beams in the lobe-plasmasheet boundary^16–19^ and those seen in dipolarization fronts^20,21^ are not fully understood, although they are thought to be a consequence of the reconnection process. Since the beams are observed even long after reconnection, their physical origin and how they may relate to the overall boundary layer and their causal connection to the global conditions remain nebulous. The Vlasov formulation as discussed below clarifies this and motivates future missions with more precise measurements.

## Theory

We first consider a pressure gradient across the magnetic field and assuming the particle orbits are not chaotic we construct a distribution function using the fact that any function of the constants of motion is a solution to the Vlasov equation. The relevant constants of motion are the guiding center position, ${X}_{g}=x+{v}_{y}/{{\rm{\Omega }}}_{\alpha }$, and the Hamiltonian, ${H}_{\alpha }(x)={m}_{\alpha }{v}^{2}\mathrm{/2}+{q}_{\alpha }{{\rm{\Phi }}}_{0}(x)$, where ${{\rm{\Phi }}}_{0}(x)$ is the electrostatic potential, and the corresponding solution is

1 $${f}_{0\alpha }({X}_{g\alpha },{H}_{\alpha }(x))=\frac{{N}_{0\alpha }}{{(\pi {v}_{t\alpha }^{2})}^{\mathrm{3/2}}}Q({X}_{g\alpha })\exp (-\frac{{H}_{\alpha }(x)}{\kappa {T}_{\alpha }}).$$

The magnetic field is in the z (north-south) direction and the pressure gradient is normal to the magnetic field in the x (earthward) direction. The subscript *α* represents the species, ${v}_{t\alpha }$ is the thermal velocity, $\kappa$ is the Boltzmann constant, $\kappa {T}_{\alpha }={m}_{\alpha }{v}_{t\alpha }^{2}\mathrm{/2}$ is the temperature away from the layer, and ${Q}_{\alpha }$ is the distribution of guiding centers designed to produce the density gradient across the layer given by,

2 $${Q}_{\alpha }({X}_{g\alpha })=\{\begin{array}{cc}{R}_{\alpha } & {X}_{g\alpha } < {X}_{g1\alpha }\\ {R}_{\alpha }+({S}_{\alpha }-{R}_{\alpha })(\frac{{X}_{g\alpha }-{X}_{g1\alpha }}{{X}_{g2\alpha }-{X}_{g1\alpha }}) & {X}_{g1\alpha } < {X}_{g\alpha } < {X}_{g2\alpha }\\ {S}_{\alpha } & {X}_{g\alpha } > {X}_{g2\alpha }\end{array}$$

${N}_{0\alpha }{R}_{\alpha }$ and ${N}_{0\alpha }{S}_{\alpha }$ are the densities in the high and low-pressure regions respectively. The quantity $|{S}_{\alpha }-{R}_{\alpha }|$ is proportional to the pressure difference between these regions and $|{X}_{g2\alpha }-{X}_{g1\alpha }|$ represents the distance over which the pressure changes. These quantities determine the magnitude and the scale-size of the electrostatic potential, which in turn determines the characteristics of the emissions that are excited at the boundary^15,22^. The values of the parameters *R*_*α*_, *S*_*α*_, *X*_*g*1*α*_, and *X*_*g*2*α*_ are model inputs determined from observations.

The density structure within the boundary layer is obtained in terms of the electrostatic potential as the zeroth moment of the distribution function, Eq. (1),

3 $${n}_{0\alpha }(x)\equiv \langle {f}_{0\alpha }\rangle =\int {f}_{0\alpha }(\overrightarrow{{\bf{v}}},\,{{\rm{\Phi }}}_{0}(x))d\overrightarrow{{\bf{v}}}={N}_{0\alpha }\frac{({R}_{\alpha }+{S}_{\alpha })}{2}\exp (-\frac{e{{\rm{\Phi }}}_{0}(x)}{\kappa {T}_{\alpha }}){I}_{\alpha }(x)$$

where,

4 $$\begin{array}{c}{I}_{\alpha }(x)=1\pm (\frac{{R}_{\alpha }-{S}_{\alpha }}{{R}_{\alpha }+{S}_{\alpha }})(\frac{1}{{\zeta }_{1\alpha }-{\zeta }_{2\alpha }})\times \\ \{{\zeta }_{2\alpha }{\rm{erf}}({\zeta }_{2\alpha })-{\zeta }_{1\alpha }{\rm{erf}}({\zeta }_{1\alpha })+\frac{1}{\sqrt{\pi }}[\exp (-\,{\zeta }_{2\alpha }^{2})-\exp (-\,{\zeta }_{1\alpha }^{2})]\},\end{array}$$

*erf* is the error function, ${\zeta }_{\alpha \mathrm{1,2}}={{\rm{\Omega }}}_{\alpha }(x-{X}_{g\alpha \mathrm{1,2}})/{v}_{t\alpha }$, and ± refers to the species charge. The quasi-neutrality condition then determines ${{\rm{\Phi }}}_{0}(x)$, which in the limit that the Debye length is smaller than the plasma scale length (which is well satisfied here) is equivalent to solving Poisson’s equation. The existence of the electric field reflects the strong spatial variability and nonlocal interactions that exist across the magnetic field due to the difference in the electron and ion distributions with their spatial variations. With Φ_0_ determined the distribution function is fully specified and higher moments can be obtained. This distribution function and the self-consistent electrostatic potential satisfy the Vlasov-Poission system and is similar to the the BGK^23^ class of solutions.

In a dipolar geometry the local values of the magnetic field as well as other plasma parameters vary with position, *s*, along the magnetic field. Hence ${{\rm{\Phi }}}_{0}\equiv {{\rm{\Phi }}}_{0}(B(s),n(s),T(s))$ becomes a function of the local parameters making it a two-dimensional function, which is harder to evaluate analytically as a nonlocal solution. However, in a dipolarization front the spatial variation scale size across the magnetic field $L\sim O({\rho }_{i})$, where ${\rho }_{i}$ is the ion gyro-radius, is much smaller than either the scale size along the magnetic field, ${L}_{\parallel }\sim {(\partial lnB(s)/\partial s)}^{-1}$ or in the dawn-dusk direction orthogonal to both^24^, which simplifies the analysis considerably by allowing the assumption of weak coupling between the physics perpendicular to the magnetic field and other orthogonal directions. Hence, to leading order Φ_0_ may be calculated locally at a given position along the field but non-locally across it.

## Results

**Figure 1:**
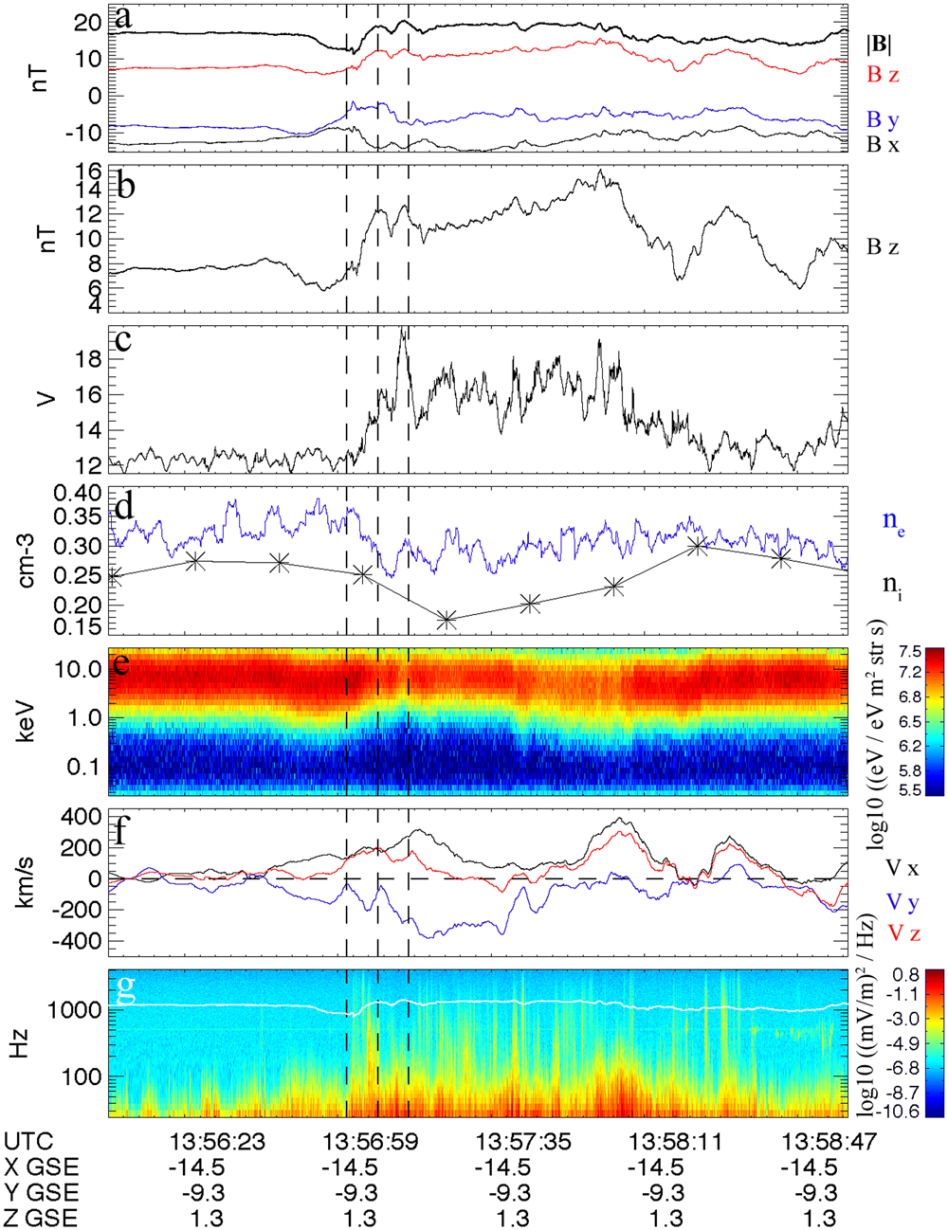
MMS fields and particle data during a dipolarization front. (**a**,**b**) Low-frequency magnetic field data (128 Samples/s) in GSE coordinates. (**c**) Spacecraft floating potential (**d**) Electron and proton density. (**e**) Electron omni-directional energy flux. (**f**) Plasma flow velocity components, in GSE coordinates, estimated from **E** × **B** measurements. (**g**) Electric field wave power spectral density.

We examined data from the MMS mission^2^ to find a case of weak compression with low ${\beta }_{e}(\,\equiv \,8\pi n\kappa {T}_{e}/{B}^{2})$ in order to minimize the electromagnetic corrections and keep the analysis tractable. Larger *β*_*e*_ cases will be considered in the future. The dipolarization front studied here was observed by MMS on 16 May 2017, near 13:56:59 UTC. Figure 1 shows the wave and particle data surrounding the front as observed by MMS 2. Comparisons between data and theory presented here use MMS 2 data, but the results are not significantly different when data from the other MMS spacecraft are used.

Figure 1a shows the x,y,z GSE components of the fluxgate magnetometer data^25^ at 128 samples/s. The bold black line shows the magnetic field magnitude. Figure 1b shows the *B*_*z*_ component in more detail to demonstrate the magnetic dipolarization that defines this event. Figure 1c shows the spacecraft potential. The Active Spacecraft Potential Control investigation^26^ was not on during this event. The increase in spacecraft potential indicates a drop in plasma density associated with the dipolarization. A density drop lowers the electron thermal current to the spacecraft surface, driving the spacecraft more positive to reach current balance with the ambient plasma^27^. Figure 1d shows the electron (*n*_*e*_) and proton (*n*_*i*_) densities. Data from the Fast Plasma Investigation (FPI)^28^ only are used to determine *n*_*e*_, while *n*_*i*_ is determined by combining density moments from FPI (up to ∼28 keV) and the Energetic Ion Spectrometer (EIS)^29^ (>30 keV). The combination techniques used here necessitate a time resolution of 1 spin (∼20 seconds). This combination is required for protons as the plasma sheet proton energy flux distribution peaks between the FPI and EIS energy ranges (near 30 keV). The electron energy distribution function is almost entirely within the FPI energy range (Fig. 1e), so combining electron density moments across instruments is not required. Figure 1f shows the three components of the plasma flow velocity in GSE coordinates, derived from **E** = −(**v** × **B)** using 32 sample/s FIELDS data^30^, smoothed using a 1/4 spin boxcar window. Figure 1g shows a spectrogram of the electric field wave data recorded by FIELDS. Shown is the sum of power spectral densities derived from all three components of the electric field time-series data. The white line indicates the local electron cyclotron frequency.

The black dashed lines in all panels of Fig. 1 indicate the spatial profile of the front used in the model calculations. This event was selected as a dipolarization front based on: the sharp increase in *B*_*z*_, collocated with a decrease in plasma density, and an Earthward flow velocity enhancement^10,31^, as well as the sudden onset of broadband wave activity^32^. This dipolarization front is located approximately -14.5 Earth radii down-tail, offset by approximately 9.3 Earth radii in the -y GSE direction and close to the equatorial plane and has a density drop of about 25% across the front.

The data are measured as a function of time in the spacecraft frame. Using the four MMS spacecraft a minimum variance analysis was used to determine the normal vector of the front. Then based on timing between the four satellites we estimated the velocity of the front to be 225 ± 8 km/s, we then used a Galilean transformation (assuming a stationary spacecraft) to convert time to distance. Because the proton flux distribution is cut off on the FPI instrument an accurate proton temperature measurement cannot be made. However, we consider the FPI proton temperature moment to be a lower bound and thus take a proton temperature of 4.7 KeV for model calculations. We estimate a mean electron temperature of 0.7 keV. Furthermore, we used a smaller window of 5 seconds before the front passed to estimate a magnetic field of ${B}_{0}$ = 13 nT (which gives an electron beta of about 0.6). With these estimates we can normalize the distance across the front to the ion gyroradius.

**Figure 2:**
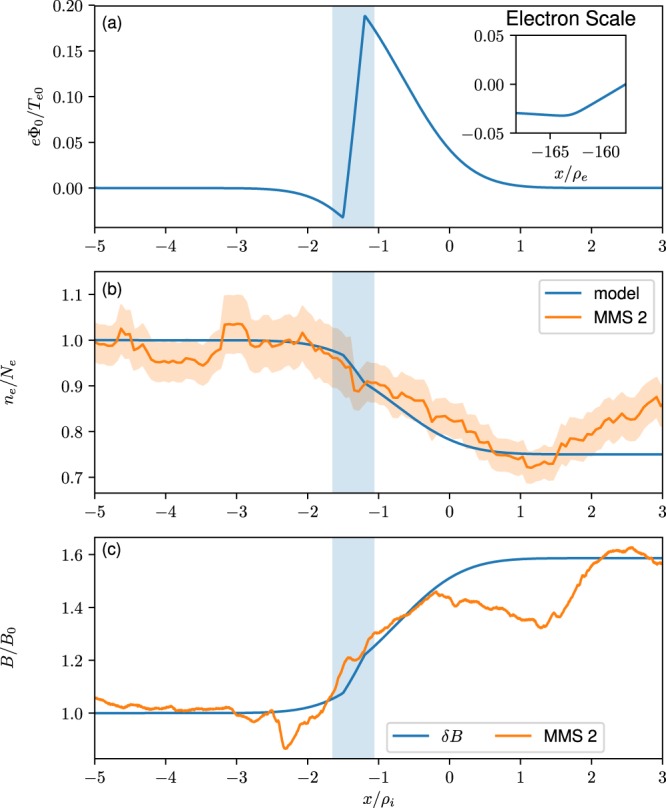
(**a**) Self-consistent electrostatic potential, Φ_0_, across the dipolarization front. The abrupt changes in value are due to variation on the electron scale. The inset shows stronger variation on electron scale around $\bar{x}\sim -\,1.5$ in the electron layer. (**b**) Self-consistent model plasma density across the dipolarization front (blue) compared with the electron plasma density (orange) from the measurements. (**c**) Magnetic flux pile up (blue) due to the cross-field current generated by the model compared to the measured magnetic perturbation (orange).

**Figure 3:**
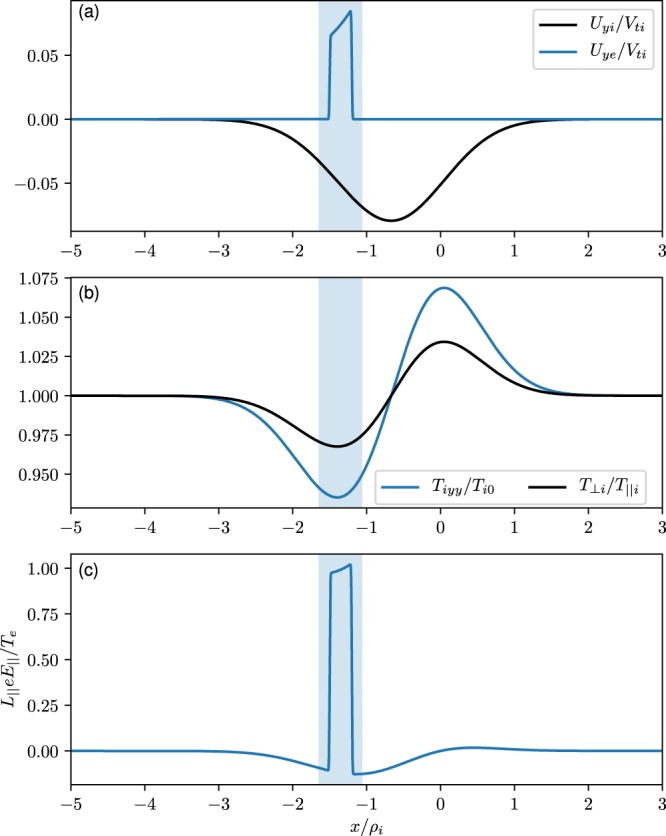
Model-calculated equilibrium features across the magnetic field of the dipolarization front considered. The blue shading shows the location of the electron layer. (**a**) Ion (black) and electron (blue) flow velocities normalized by the ion thermal velocity. (**b**) Ion temperature component in the y-direction (blue) and anisotropy ratio (black). (**c**) In the dipolar magnetic field geometry an electric field component is generated along the magnetic field lines.

Figure 2 shows the model calculation for this event. Figure 2a is a plot of the electrostatic potential that develops across the magnetic field when quasi-neutrality is enforced. Here $\bar{x}=x/{\rho }_{i}$, ${\rho }_{i}$ evaluated in the Earthward region outside the layer. We use ion ${X}_{g1i}=-\,1.0{\rho }_{i}$ and ${X}_{g1e}=-\,1.5{\rho }_{i}$ while electron ${X}_{g2i}=-\,0.5{\rho }_{i}$ and ${X}_{g2e}=-\,1.2{\rho }_{i}$ with ${R}_{i,e}=1.0$ and ${S}_{i,e}=0.75$. The potential is localized over the boundary layer and has both positive and negative slopes but with different scale sizes. Consequently, the corresponding electric field will have both negative and positive components with different magnitudes. The model parameters were chosen so that the density profile was consistent with the electron density measurement, and the remaining quantities were computed self-consistently. The density is plotted in Fig. 2b on top of the data. We find that with the estimated ion temperature the density gradient scale length is of the order of the ion gyroradius, although the gradient is steeper around $\bar{x}\sim -\,1.5$, shaded in blue, where the variation in the potential is strongest. We note that if the ion temperature is higher (as expected) the gyroradius would be larger, which would create a larger ambipolar electric field, more kinetic-scale features of the equilibrium, and more free energy to drive waves. The self-consistent electron and ion flows are unequal as described in Fig. 3a and the resulting current across the boundary layer results in magnetic flux pile up shown in Fig. 2c, which is in agreement with observation.

Figure 3a shows the self-consistent y component of the electron flow (blue) centered around $\bar{x}\sim -\,1.5$ and ion flow (black) centered around $\bar{x}\sim -\,0.75$ normalized by the ion thermal velocity. The electron and ion flows peak at different locations and are oppositely directed. This is due to the unique distribution function that develops self-consistently in the layer. The separation of electron and ion layers is a kinetic effect and was observed in the plasmasheet-lobe interface^33^. The electron flow is essentially the **E**(*x*) × **B** flow modified by the inhomogeneity. The origin of the electric field is ambipolar due to the difference in the electron and ion gyro-radii in balance with the Lorentz force and saturates as the pressure gradient scale size reduces below *ρ*_*i*_. The ions remain magnetized and execute **E**(*x*) × **B** drift albeit with different magnitude and character than the electrons^34^ as elaborated in Sec. IV. This is unlike the electron magnetohydrodynamic (eMHD) treatment in which the ions are treated differently and the electric field is inversely proportional to the pressure gradient scale size.

**Figure 4:**
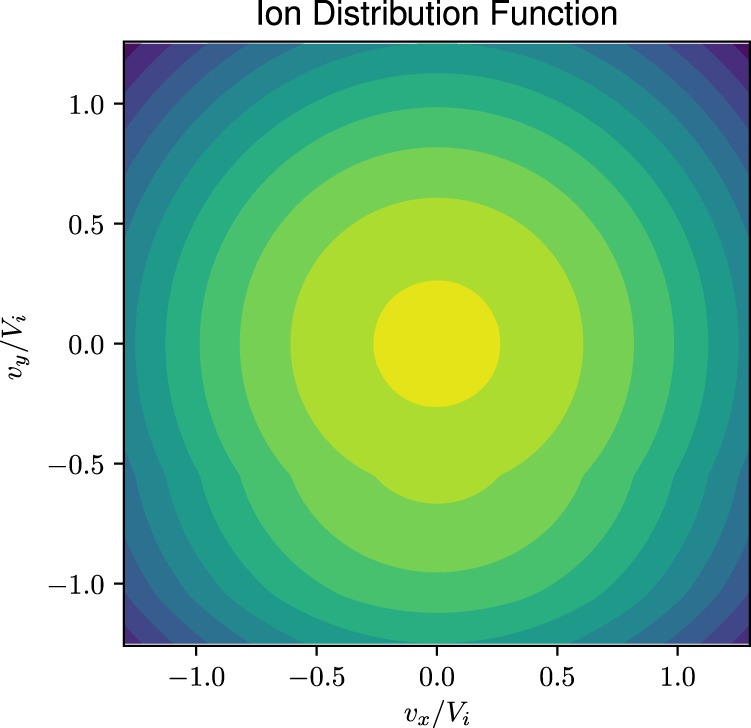
The presence of a non-uniform transverse electric field makes ion and electron distribution functions in the dipolarization front non-gyrotropic. The non-gyrotropy is more visible in the ion distribution function than in electrons. The distribution function is plotted at $x/{\rho }_{i}=0.0$.

Figure 3b is the variations of ion temperature in the y direction. There is no similar variation in the x temperature. Consequently, an anisotropy develops across the layer in the perpendicular temperatures (${T}_{\perp }={T}_{xx}+{T}_{yy}$). The asymmetry in temperatures in the x and y directions is an unusual feature that originates due to the gradient in ${E}_{\perp }(x)$ and makes the distribution non-gyrotropic as shown in Fig. 4 and also causes temperature anisotropy between the perpendicular and parallel directions. There is similar effect on the electron temperature but the magnitude is smaller.

Figure 4 is a plot of the ion distribution function, which is non-gyrotropic. The electron distribution is also non-gyrotropic but the asymmetry between the x and y components is milder than that of the ions. This is due to the difference in the ratio of the **E** × **B** velocity gradient and the gyro-frequency (i.e., $(d{V}_{E\alpha }/dx)/{{\rm{\Omega }}}_{\alpha }$) of the species as discussed below.

The physics becomes more transparent if we consider the weak gradient limit defined by $\varepsilon \equiv {\rho }_{\alpha }/L < 1$ but but $\eta (x) > 0$ in which the distribution function may be simplified to^35^,

5 $${f}_{0}\simeq \frac{{n}_{0}}{\sqrt{\eta (x)}{(\pi {v}_{t}^{2})}^{\mathrm{3/2}}}\exp (-({v}_{x}^{2}+{({v}_{y}-{V}_{E}(x))}^{2}/\eta (x)+{v}_{z}^{2})/{v}_{t}^{2})+O(\varepsilon )$$

where $\eta (x)=1+(d{V}_{E}(x)/dx)/{\rm{\Omega }}$, and ${V}_{E}(x)={\langle -c{E}_{x}(x)/B\rangle }_{g}$ is the gyro-averaged **E**(*x*) × **B** drift. If $d{V}_{E}(x)/dx\to 0$ Eq. (5) reduces to a Maxwellian, which is a stable distribution. This shows that global compression results in a deviation from Maxwellian through the velocity gradient, which is a source of free energy for waves. Thus, in the collisionless environment compression triggers a relaxation mechanism to reach a steady state through the emission of waves, which dissipates the velocity gradient. This distribution was used in particle-in-cell (PIC) simulation of boundary layers to demonstrate the relaxation process^36^. The dependence on the spatial gradient of the flow through the parameter *η* and its asymmetric appearance in the distribution function is noteworthy. It explains why the temperature in the y direction is preferentially affected by the localized electric field across the magnetic field in the x direction. It also explains the asymmetry in the *v*_*x*_ and *v*_*y*_ integrations, which breaks the gyrotropy and introduces an effective anisotropy in the temperature in the x and y directions.

The stability of this equilibrium was analyzed and found to support a hierarchy of instabilities in the frequency range starting from below the ion gyro-frequency to above the electron gyro-frequency depending on the electric field gradient, which is also defined as the shear frequency ${\omega }_{s}\equiv d{V}_{E}/dx$^22^. PIC simulations^37,38^ and laboratory experiment^39^ show that these instabilities can cumulatively constitute a broadband spectral signature.

As we move along the magnetic field the x and z coordinates rotate by an angle $\theta$. Since the local values of the magnetic field and other plasma parameters are different, the electrostatic potential will be different giving rise to an electric field along the magnetic field direction. Since ${{\rm{\Phi }}}_{0}\simeq {{\rm{\Phi }}}_{0}(B(s))$, the parallel electric field is ${E}_{\parallel }(s)\equiv -\,\partial {{\rm{\Phi }}}_{0}(B(s))/\partial s=(x/{L}_{\parallel }){E}_{x}(x)$. Figure 3c shows that *E*_||_ peaks in the electron layer and varies in x. Non-thermal plasma particles subjected to *E*_||_ will be accelerated along the magnetic field to form inhomogeneous beams or flows. The generation of the beam along the field line by this process provides the physical basis for a non-reconnection origin of the observed beams and its causal connection to the global compression.

The stability of inhomogeneous parallel flows has also been analyzed both theoretically and through laboratory experiments. Like its transverse counterpart the spatial gradient in the parallel flow can also support a hierarchy of oscillations^40–48^. Cumulatively the gradient in the parallel and perpendicular flows, which arises naturally as a consequence of the global compression, constitute a rich source for waves in a broad frequency and wave vector band and their emission is necessary to relax the stress, i.e. gradients, that builds up in the layer during the dipolarization process. In particular, the generation of both electrostatic and electromagnetic waves around the lower hybrid frequency by velocity gradient has been extensively studied^15,46,49–53^. Simulations^37,38^ indicate that these waves produce anomalous viscosity and relax the gradients to reach a steady state. Due to the strong spatial gradient in the dipolarization fronts across the magnetic field the plane wave or WKB approximations will break down. Hence, these waves must be treated as an eigenvalue problem in the stationary dipolarization front frame.

Existence of *E*_||_ indicates that the off-diagonal terms of the pressure tensor, ${\overleftrightarrow{P}}_{\alpha }={m}_{\alpha }\int (\overrightarrow{v}-\overrightarrow{V})(\overrightarrow{v}-\overrightarrow{V}){f}_{0\alpha }d\overrightarrow{v}$, are non-zero and are necessary to balance it in equilibrium, i.e.,

6 $$en(x){E}_{\parallel }=-\,(\nabla \cdot {\overleftrightarrow{P}}_{\alpha }(x))\cdot \hat{s}=-\,({\partial }_{x}{p}_{xx}{\hat{b}}_{x}+{\partial }_{x}{p}_{xz}{\hat{b}}_{z})$$

where ${\hat{b}}_{x}=\,\sin (\theta )$ and ${\hat{b}}_{z}=\,\cos (\theta )$, and to leading order $\partial /\partial y=\partial /\partial z\to 0$ because the spatial variation is strongest in the x direction at a given location along the magnetic field. Away from the equatorial plane the pressure gradient diminishes which reduces the electric fields in both parallel and perpendicular directions.

## Discussion

The complex attributes of the boundary layer equilibrium are caused by the highly localized ambipolar electric field that develops across it in response to the global compression and are essentially kinetic. Our analysis of the physics in this highly inhomogeneous environment is based on moments of particle distributions that develop self-consistently with the boundary layer variations and quasi-neutrality where the individual particle dynamics is averaged through velocity integration over the distribution. However, for further insight it is instructive to consider individual particle orbits in such an environment. For a weak electric field gradient, i.e., ${\rho }_{/}L < 1$ and $\eta  > 0$, the equation of motion is ${\dot{v}}_{x}=\eta (x){\rm{\Omega }}{u}_{y}+O({\varepsilon }^{2})$ and ${\dot{u}}_{y}=-\,{\rm{\Omega }}{v}_{x}$ where ${u}_{y}=({v}_{y}-{V}_{E}(x))/\eta (x)+O({\varepsilon }^{2})$^35^. Combining them we get ${\ddot{v}}_{x}=-\,\eta (x){{\rm{\Omega }}}^{2}{v}_{x}+O({\varepsilon }^{2})$, which indicates that to leading order the electric field gradient affects the particle orbit through $\eta (x)$ by renormalizing the gyro-frequency ${\rm{\Omega }}\to {\rm{\Omega }}^{\prime} \equiv \sqrt{\eta (x)}{\rm{\Omega }}$. Depending on the sign of the gradient the effective gyro-radius, $\rho ^{\prime} \equiv {v}_{th}/{\rm{\Omega }}^{\prime}$, can be larger or smaller, which will be reflected in the averaged equilibrium quantities as larger or smaller temperatures. The variation in temperature, however, is a result of the velocity gradient and not velocity randomization.

In the weak gradient limit, the higher-order derivatives of the electric field are not important and are lumped into *O*(*ε*^2^) in the equations but they become critical for strong gradients. For strong gradient $\eta  < 0$ and ${\ddot{v}}_{x}=|\eta (x)|{{\rm{\Omega }}}^{2}{v}_{x}+O({\varepsilon }^{2})$, which indicates that the restoring nature of the force becomes divergent and the particle accelerates along the electric field. Gavrishchaka^34^ studied the strong gradient limit. He showed that for strong gradient multiple guiding centers can arise and the particles do not accelerate indefinitely unless the electric field is linear, which is a pathological case. Higher order derivatives prevent indefinite linear acceleration, which results in modified orbits that are no longer the ideal gyro-motion. The particle acquires an effectively larger gyro-radius around a new guiding center.

The strong gradient condition is met around $\bar{x}\sim -\,1.5$ for individual ions because $|d{V}_{E}/dx|\gg {{\rm{\Omega }}}_{i}$, which will accelerate the ions along x. This effectively results in a larger gyro-radius (${\rho ^{\prime} }_{i}\gg L$). Consequently, the ions sample the electric field only over a fraction of their larger gyro orbit resulting in a lower average **E**(*x*) × **B** drift thereby reducing the velocity gradient such that *η* becomes positive. Consequently, the average ion **E**(*x*) × **B** flow for the ion distribution is small and results in average ion $\eta  < 1$ but positive. For an individual electron, however, $|d{V}_{E}/dx| < {{\rm{\Omega }}}_{e}$ keeping electron *η* always positive.

When particles execute **E** × **B** drift, the Lorentz force can balance the transverse electric field and the existence of a pressure gradient is not a necessary condition. Large localized transverse electric fields can be maintained in quasi-neutral plasma with little density gradient as found in the auroral region^54^ and shown theoretically in the weak gradient limit^35^. Because the electrons experience a weak gradient condition the electron *η* remains positive and their orbits are not much affected. But the ions near $\bar{x}\sim -\,1.5$ experience a strong gradient and their orbits are substantially affected. They effectively acquire a larger gyro-radius and experience reduced electric field but remain magnetized. This results in separate electron and ion layers. Therefore, the traditional eMHD notion of the dynamics and ion-electron interactions in a dipolarization front^3^ may have to be revised.

## Conclusions

In the preceding analysis we showed that dipolarization fronts are narrow boundary layers consisting of electron and ion layers with strong spatial variations in velocity as well as temperature and pressure anisotropy. These are generated by the self-consistent ambipolar electric field resulting from global compression. Dipolarization fronts are dynamic regions characterized by plasma distributions that are far from thermodynamic equilibrium in which the stress (i.e., velocity gradient) generated by global compression constantly adjusts to seek a lower energy state through relaxation. In a collisionless environment the relaxation is achieved through the emission of waves that allows a steady state to be reached. For this purpose the waves must be generated through dissipation of the free energy source of the dipolarization front itself and not imported from outside. The global compression is also responsible for an electric field component along the magnetic field direction, which can produce parallel beams and flows that are unrelated to the reconnection process.

Capturing the small-scale details of a DF in global kinetic simulations^5–8^ of the magnetic reconnection process is not practical. Global scale simulations are still limited by artificially small mass ratios and insufficient particles per cell to accurately resolve ion and electron gyro scale variations for ambipolar effects, which our kinetic analysis shows to be significant. More importantly, a global scale kinetic simulation must start from realistic initial condition and be subject to realistic boundary conditions. Because of the sparse nature of observing satellites both of these conditions are poorly defined leading to simulations that may not reflect the actual global response of the system. Hence, we address the small (dissipation) scale dynamics of a DF by using local kinetic analysis in the DF frame, which is anchored in reality by requiring that the distribution function reproduce the observed density and magnetic field profiles reasonably well.

Our goal has been to keep the analysis simple in order to develop an insight into the physical underpinnings of the kinetic equilibrium of a dipolarization front. We restricted the analysis to the neighborhood where the pressure gradient across the magnetic field remains approximately constant along the magnetic field. However, on larger scale the pressure gradient will decrease along the magnetic field gradually, which will diminish the electrostatic potential until it becomes negligible. This effect is not included in the model. We also restricted the analysis to low *β*_*e*_ in order to ignore the correction of the magnetic flux pile up on the equilibrium. Higher *β*_*e*_ will be subsequently considered. Perhaps more importantly, this equilibrium model does not include the feedback effects of the waves, which relax the initial gradients in PIC simulations of the plasmasheet-lobe interface^37,38^. Since satellites generally observe a steady (saturated) state the observed parameters are relaxed from their initial values. Thus, for more accurate comparison with the data the stability of the equilibrium discussed here will be examined and will be discussed in a future article.
